# Phonetic Effects in the Perception of VOT in a Prevoicing Language

**DOI:** 10.3390/brainsci12040427

**Published:** 2022-03-23

**Authors:** Viktor Kharlamov

**Affiliations:** Department of Languages, Linguistics and Comparative Literature, Florida Atlantic University, Boca Raton, FL 33431, USA; vkharlamov@fau.edu

**Keywords:** prevoicing, VOT, aerodynamic voicing constraint, perceptual identification, Russian

## Abstract

Previous production studies have reported differential amounts of closure voicing in plosives depending on the location of the oral constriction (anterior vs. posterior), vocalic context (high vs. low vowels), and speaker sex. Such differences have been attributed to the aerodynamic factors related to the configuration of the cavity behind the oral constriction, with certain articulations and physiological characteristics of the speaker facilitating vocal fold vibration during closure. The current study used perceptual identification tasks to examine whether similar effects of consonantal posteriority, adjacent vowel height, and speaker sex exist in the perception of voicing. The language of investigation was Russian, a prevoicing language that uses negative VOT to signal the voicing contrast in plosives. The study used both original and resynthesized tokens for speaker sex, which allowed it to focus on the role of differences in VOT specifically. Results indicated that listeners’ judgments were significantly affected by consonantal place of articulation, with listeners accepting less voicing in velar plosives. Speaker sex showed only a marginally significant difference in the expected direction, and vowel height had no effect on perceptual responses. These findings suggest that certain phonetic factors can affect both the initial production and subsequent perception of closure voicing.

## 1. Introduction

The current study examined the link between production and perception of voicing in initial voiced plosives in Russian, a prevoicing language. In the domain of production, the amount of closure voicing is known to vary depending on the plosive’s place of articulation, its vocalic context, and speaker’s biological sex. This research aimed to determine whether similar asymmetries exist in perception, with listeners expecting differential amounts of voicing for anterior versus posterior plosives, low versus non-low vowel contexts, and female versus male voices.

### 1.1. Aspirating vs. Prevoicing Languages

The contrast between voiced versus voiceless plosives involves a timing difference in the onset of vocal fold vibration relative to the release burst of the plosive. This is known as ‘voice onset time (VOT)’ [1,2,3]. In languages such as English and German, both voiced and voiceless plosives are usually articulated with a positive VOT in initial positions, meaning that the onset of voicing occurs after the release burst. The voicing lag is short for voiced segments (approximately 30 ms or less) and long for voiceless segments (usually in excess of 50 ms) [4]. The long-lag VOT is achieved by aspirating the plosive, so such languages are referred to as ‘aspirating’.

A different VOT pattern is seen in the so-called ‘prevoicing’ languages, including Dutch and Russian, where voiced initial plosives are produced with negative VOT [5,6,7]. This means that voicing starts during closure, and the VOT lead in voiced plosives is usually contrasted with zero or short lag VOT in voiceless plosives that are articulated without aspiration in initial positions. The rates of prevoicing can differ across languages and dialects. Continental French and Russian, for example, have robust prevoicing in initial plosives, with negative VOT found in over 94% of voiced tokens [6,7,8,9]. In Spanish, the rate of prevoicing has been reported to be around 86% [9]. In Dutch, negative VOT has been observed in 75% of initial voiced plosives only [5]. There are also languages such as Polish that contrast prevoiced versus (slightly) aspirated plosives [10] as well as languages with optional prevoicing. For example, although English is not a prevoicing language, voicing lead does occur in some speakers and under certain conditions, such as hyperarticulated and laboratory-type speech [4,11,12]. Prevoicing is also common in African American English [13] and in some regional dialects, including Inland California [14].

### 1.2. Production of Voicing

According to the myloelastic-aerodynamic theory of voice production, voicing is achieved by keeping the vocal folds adducted and allowing air to flow through the glottis, which causes vocal fold vibration [15]. To maintain the airflow, subglottal pressure needs to be higher than the pressure inside the oral cavity. This is difficult to implement for plosives, since they are articulated with a period of complete oral closure that leads to rapid equalization of subglottal and supraglottal pressure. This phenomenon is known as the ‘aerodynamic voicing constraint (AVC)’ [16,17,18]. Speakers use a variety of mechanisms to overcome the AVC and facilitate the production of voicing, such as lowering the tongue body to enlarge the vocal tract [19] or allowing nasal venting during oral closure [7,9].

The AVC is especially problematic for utterance-initial plosives when the subglottal pressure has not yet reached its peak level [20] as well as the more posterior places of articulation [20,21,22,23]. Anterior plosives are produced with a larger volume of space behind the oral constriction and with access to more compliant surfaces (including most of the tongue surface and the inside walls of the cheeks). This facilitates expansion of the supraglottal cavity in reaction to rising pressure. In contrast, posterior plosives are articulated with a smaller cavity behind the constriction and less compliant surfaces (e.g., the back of the tongue and the pharyngeal wall). As a result, oral pressure increases faster and the difference between oral versus subglottal pressure is neutralized sooner, which inhibits voicing.

Asymmetries in closure voicing duration or its frequency across places of articulation have been noted in several previous production studies. For example, initial velar plosives in English show up to 14ms more prevoicing (when produced with negative VOT) [4]. English velars are also more likely to be devoiced than non-velars [24]. In Polish, velar plosives have up to 22 ms less prevoicing than bilabials and alveolars [10]. In Dutch, bilabial plosives have a higher rate of prevoicing than alveolars (there is no voiced velar plosive in the language), and there is also a non-significant durational difference in the expected direction (possibly due to a small sample size) [5]. In Swedish, there is up to 30 ms less prevoicing for velars than non-velars [25]. In Russian, there is between 12 ms to 18 ms less prevoicing in velar plosives compared to bilabials and alveolars [6,7].

In addition to being affected by the plosive’s place of articulation, closure voicing may vary depending on adjacent vowel height, with anticipatory coarticulation affecting the size of the cavity behind the oral constriction and influencing the extent to which air pressure can be lowered [23,24]. However, production findings for the role of vowel height have been less consistent than the effects of consonantal posteriority. Some studies report that vocal fold vibration is easier to produce for plosives followed by high vowels, which may be attributed to a larger pharyngeal cavity during the articulation of high vowels [23,24,26]. Other studies show a general faciliatory effect of adjacency to a vowel but no differences between high versus low vowels [5]. In Russian, the effect of vowel height is in the opposite direction, with up to 12ms more prevoicing seen for plosives followed by non-high vowels [7]. Such a finding may be due to greater exposure of cheek walls for non-high vowels [23] or greater vocal fold tension for high vowels [27].

One more factor known to affect closure voicing is speaker’s biological sex (usually referred to as ‘gender’ in previous studies) [5,6,13,25]. Male speakers tend to have larger vocal tracts [28], which can be expected to make it easier for males to produce and maintain voicing during complete oral occlusion. However, similarly to the effects of vowel height, the findings for speaker sex have been inconsistent. In Dutch, prevoicing is more frequent in male speakers (21% difference) and closure voicing is also longer for males (by 20 ms) but the durational difference is not significant (which may be due to a small sample size of five speakers per sex) [5]. In Norwegian, the effect of speaker sex is reversed, with longer and more frequent prevoicing found in female tokens, which may be related to female speech being more hyperarticuled [29]. In Russian, the speaker sex effect is either not significant (possibly due to averaging across places of articulation) [8] or it is a general effect, with male speakers producing 10 ms longer prevoicing [6], or there is no overall difference but there is an interaction between speaker sex and vocalic context, with Russian males producing up to 22 ms less prevoicing in tokens with a high vowel [7].

Overall, previous production studies have shown that the likelihood of closure voicing and its duration vary across places of articulation, with stronger prevoicing seen for the more anterior plosives. Voicing also appears to be sensitive to the quality of the following vowel but the direction of the effect is not consistent in production, with some studies showing facilitation and other studies reporting inhibition of prevoicing in high vowel contexts. Finally, speaker sex may also play a role, with male speakers producing more frequent or longer closure voicing; however, the effect of speaker sex is also limited and not always consistent in production.

### 1.3. Perception of Voicing

While the production side of voicing has now been examined for several prevoicing languages, little is currently known about the way listeners perceive negative VOT and whether perceptual judgments are affected by the voicing asymmetries that exist in production. Most previous perception studies have concentrated on aspirating languages, such as English, and the general finding has been a typical categorical perception S-curve with a cross-over in perceptual judgments from ‘voiced’ to ‘voiceless’ occurring between +25 ms to +50 ms [10,30,31]. When two or more places of articulation are examined, positive VOT usually shows an effect of posteriority. In English, the cross-over boundary occurs up to 17ms later for velars than non-velars [31]. Speakers who are bilingual in English and a prevoicing language, such as Spanish, also show cross-over boundaries in the positive range as well as the expected posteriority effect of up to 10 ms [32].

For prevoicing languages, most previous perception studies only examined one place of articulation. Cross-over boundaries of −4 ms and −11 ms have been reported for bilabial plosives in Puerto Rican Spanish and Peruvian Spanish, respectively [33]. In Hebrew, which contrasts prevoiced versus (slightly) aspirated stops, perceptual judgments for bilabials show a category shift at around +6 to +7 ms [34]. For alveolars, Polish shows cross-over boundaries ranging from −3.5 ms to +21 ms, with the exact boundary location dependent on the range of values in the VOT continuum [10]. In Russian, the crossover point for alveolars has been observed at around −16 ms [35]. For Dutch, category boundaries are known for both bilabials (−9.5 ms) and alveolars (−3.5 ms) [5], which suggests that less voicing may in fact be needed for the more posterior plosives to be classified as voiced. However, the Dutch values were obtained in a classification tree analysis, so they may not be directly comparable to the boundaries established in perceptual identification studies mentioned above.

### 1.4. Current Study

The current research focused on determining whether the effects of place of articulation, vowel height, and speaker sex that occur in production can also be found in perception. The study examined perceptual identification responses for plosive-initial CV sequences with VOT values in the ambiguous range around the cross-over boundary. The language of investigation was Russian, a prevoicing language with very limited previous research on VOT perception. Based on the earlier production findings, the study was expected to show that if place of articulation did affect perceptual judgments, less voicing would be needed for the more posterior plosives to be identified as voiced. For vowel height, a difference in responses to tokens with high versus low follow vowels could be expected but the exact direction of the effect could not be predicted due to the inconsistency of previous production findings. For speaker sex, male tokens could be predicted to be judged as voiceless more often than female tokens with the same amount of VOT, although the magnitude of the effect was likely to be limited due to the lack of a consistent pattern in production. If supported by the actual results, such findings would provide evidence for the AVC and related phonetic factors affecting both the initial production of voicing and its subsequent perception.

## 2. Materials and Methods

### 2.1. Participants, Stimuli, and Procedures

Participants were monolingual speakers of Russian (*n* = 60; 32 female, 28 male; age range of 18 to 30, mean age of 20.4). They were recruited and tested at a university campus in Perm, Russia. During the pre-test interview, all participants indicated having beginner-level knowledge of other languages, acquired in a classroom setting, and no regular exposure to non-Russian speech. None self-reported a hearing problem or another issue that may affect speech production or perception.

The stimulus list consisted of 180 CV tokens that differed across consonantal place of articulation, following vowel height, speaker sex, and VOT level. The initial plosive was bilabial ([b/p]), alveolar ([d/t]), or velar ([g/k]). The vowel was low ([a]), mid ([o]), or high ([u]). Other vowels were not used because of the restrictions related to consonantal palatalization in Russian. The CV sequences were produced by two native Russian speakers (male, 22 y.o.; female, 20 y.o.). Depending on the token, the mean pitch ranged from 125 Hz to 130 Hz for the male speaker and between 245 Hz and 255 Hz for the female speaker. For each CV sequence, a VOT continuum was created in Praat [36]. VOT values ranged from −60 ms to +30 ms in 10 ms steps. Tokens that were originally produced with at least 60 ms of prevoicing served as the base. For negative VOT, voicing was removed in 10ms increments, starting at −60 ms. For positive VOT, bursts were extended by splicing in burst noise from CV sequences with initial voiceless plosives with the same place of articulation and the same following vowel. Intensity of the tokens and vowel duration were adjusted to match across places of articulation, vocalic contexts, and speaker sexes. A set of 10 CV sequences with initial fricatives was also prepared for use in a training module ([va, fo, zu], etc.).

**Figure 1:**
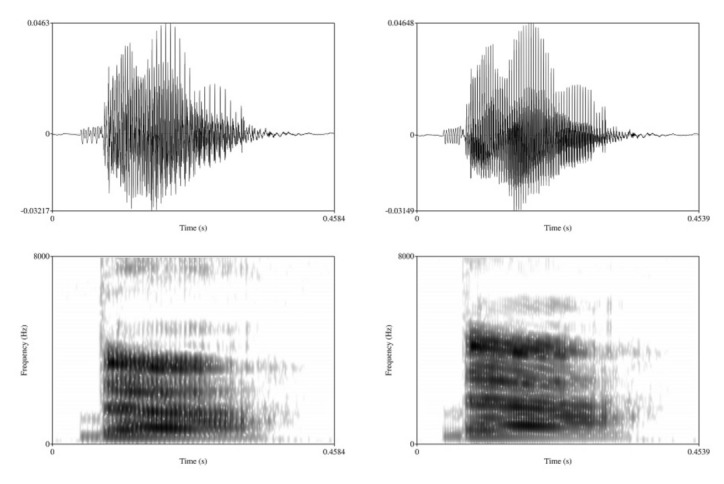
Prevoiced [da] token with −30 ms VOT. Original male voice (**left**) and resynthesized female voice (**right**).

In addition to the naturally produced female and male speech, the study used tokens with resynthesized speaker sex. This was done to ensure that differences between female versus male tokens, if observed, were not driven by acoustic properties other than VOT. For this purpose, the original items produced by the male speaker were resynthesized using the ‘Change gender’ function in Praat [36]. The new median pitch was set to 250 Hz, and formants were shifted by a ratio of 1.1, which adjusts the formants upward to better match female voice characteristics. Pilot testing showed that resynthesis of female tokens into male resulted in an unnatural-sounding voice that participants found distracting, so only the male-to-female resynthesis was used in the study. Sample tokens in original male and resynthesized female voices are provided in Figure 1.

Experimental sessions took place at a psychology lab and lasted approximately 60 min. During the session, participants performed a forced-choice identification task. The ExperimentMFC module in Praat [36] was used to present the stimuli and record listeners’ responses. Instructions were given verbally and also printed on screen. Participants were instructed to identify the sequence (e.g., whether they heard ‘ba’ or ‘pa’) and to answer as quickly and accurately as possible. The experiment started with a short training stage that utilized words with initial fricatives. This was followed by presenting experimental items in a randomized order. The tokens were presented binaurally through a pair of sound-insulating headphones. The next stimulus was presented 1 second after a response was entered. Each participant responded to a total of 900 tokens (3 places of articulation × 3 vowels × 2 speaker sexes × 10 VOT levels = 180 tokens × 5 repetitions per token = 900 tokens). The experimental script was set to pause after presenting a set of 50 items, and participants were strongly encouraged to take breaks in-between the sets. Since some CV sequences matched existing Russian words (e.g., [ta] meaning ‘that (fem.)’ or [da] meaning ‘yes’), stimuli were only referred to as ‘syllables’ throughout the experiment to help mitigate a lexical effect, and the item’s lexical status and existence of a lexical competitor were also tested as control variables in statistical modeling.

Data were collected over two experiments. In Experiment 1, participants (*n* = 30; 16 female, 14 male; age range of 18 to 29; mean age of 20.5) listened to the original voices. In Experiment 2, participants (*n* = 30; 16 female, 14 male; age range of 18 to 30; mean age of 20.4) listened to original male and resynthesized female voice tokens. Since the experiments were performed anonymously and were conducted at the same university, it is possible that some participants took part in both experiments; however, the experimental sessions were two years apart, so no carry-over effects would be expected.

### 2.2. Data Analysis

Participants’ responses were analyzed statistically in R [37]. Given the binary nature of the dependent variable (‘voiced’ versus ‘voiceless’), a logistic mixed-effects model was constructed for each experiment using the glmer function of the ‘lme4’ package [38]. Forest plots illustrating the odds ratios for the models were created with the ‘sjPlot’ package [39]. Each model examined participants’ responses to the tokens with VOT durations in the −20 ms to +20 ms range. This was the time window that encompassed the categorical shift in perception from ‘voiced’ to ’voiceless’ and the adjacent ambiguous regions. The model tested for the fixed effects for VOT duration, consonantal place of articulation, the following vowel, and speaker sex. It also included all 2-way interactions with VOT duration as well as two additional interactions that were significant in production for Russian: (i) consonantal place × vocalic context and (ii) speaker sex × vocalic context [7]. Prior to analysis, VOT duration was rescaled. Place of articulation was coded as ‘bilabial’ (yes = 1, no = 0) and ‘velar’ (yes = 1, no = 0). Vowel height was coded as ‘high’ (yes = 1, no = 0) and ‘low’ (yes = 1, no = 0). Speaker sex was coded as ‘male’ (yes = 1, no = 0).

Several control variables were also tested during the model selection process, including listener sex (‘male’; yes = 1, no = 0), listener age (in full years), lexical status of the stimulus (i.e., whether the CV sequence matches a real word of Russian; yes = 1, no = 0), and lexical competition (i.e., existence of a voicing-based minimal pair counterpart; yes = 1, no = 0). The lexical factors were coded on the basis of a comprehensive Russian thesaurus [40]. The control variables were not retained in the final model as they did not improve model fit (as indicated by ANOVAs comparing models with and without control variables).

Models with all relevant fixed effects and interactions, participants and items as random effects, and correlated random slopes did not converge, so the random effects structure was gradually simplified until maximal models with non-singular fits were found [41]. For Experiment 1, the maximal model included a random intercept for item. For Experiment 2, the maximal model had a random slope for participant by VOT duration and a random intercept for item.

## 3. Results

### 3.1. Experiment 1

Experiment 1 utilized the original voice tokens. Two fixed effects were identified as significant in the glmer model: VOT duration (β=−10.96;z=−13.48;p<0.001) and consonantal place (velar vs. non-velar; β=0.41;z=3.18;p<0.01). All other fixed effects and interactions were not significant (all *p*s > 0.1). Full results of statistical modeling are provided in Table A1 in Appendix A.

**Figure 2:**
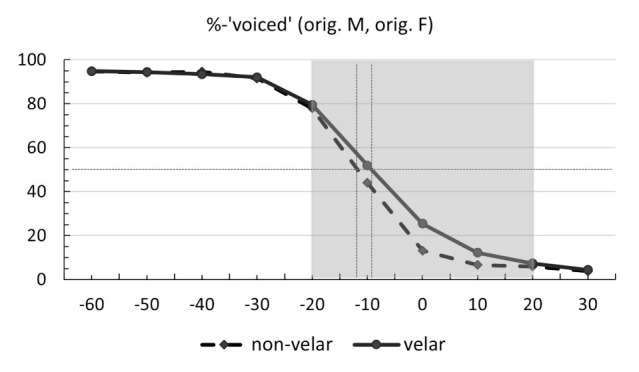
Identification curves for the original voice non-velars (dashed line) and velars (solid line) identified as ‘voiced’ across 10 VOT levels. Thin dotted lines mark the 50% boundary. Statistical modeling was conducted on the area highlighted in grey.

Figure 2 shows the identification curves for the statistically significant contrast between velars versus non-velars. Tokens with velar plosives are marked with a solid line. Items with non-velars (bilabials, alveolars) are shown with a dashed line. The y-axis represents the mean rates of ‘voiced’ responses. The x-axis shows the 10 VOT levels (from −60 ms to +30 ms in 10 ms steps). Category boundaries at 50% (cross-over points) are marked with thin dotted lines. The area highlighted in grey was used in statistical modeling. As can be seen in the figure, classic S-shaped identification functions were obtained along the VOT continuum for both velars and non-velars. As prevoicing duration decreased, the rate of ‘voiced’ responses also decreased. For non-velars, a shift in perceptual judgments from ‘voiced’ to ‘voiceless’ occurred at −12 ms. For velars, the category boundary was at −9 ms. Table A2 in Appendix A provides the full results for the mean rates of ‘voiced’ responses, including the differences across the levels of non-significant factors.

**Figure 3:**
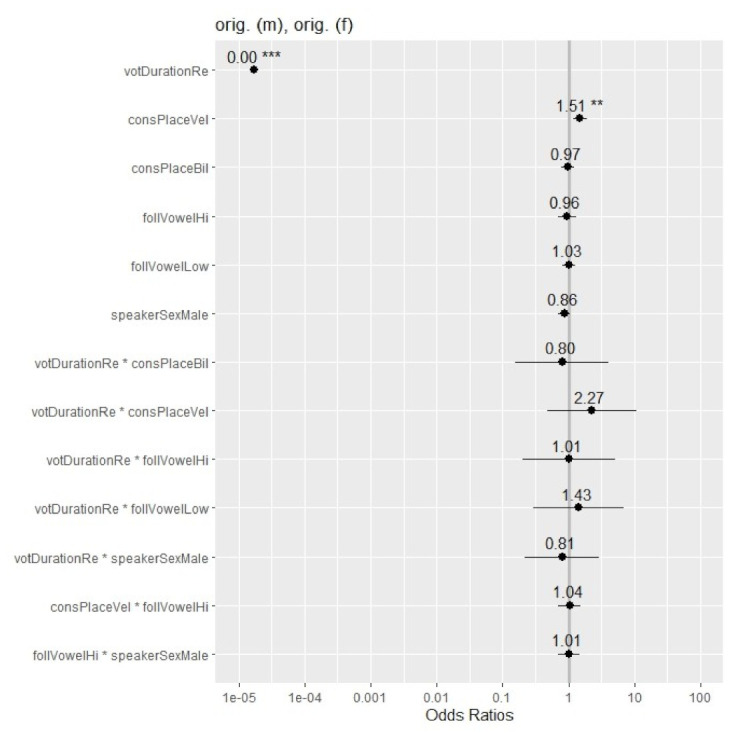
Odds ratios (ORs) of ‘voiced’ responses across the fixed effects and interactions in the glmer model for the original male and female voice tokens in the −20 ms to +20 ms VOT range. Significance is shown with asterisks (‘***’: *p* < 0.001; ‘**’: *p* < 0.01.). Confidence intervals (CIs: 95%) are marked with whiskers.

Figure 3 provides the odds ratios (ORs) for ‘voiced’ responses in the −20 ms to +20 ms VOT window. OR values represent the odds that an outcome will occur given a particular exposure, compared to the odds of the outcome occurring in the absence of that exposure [42]. The figure shows mean ORs for the fixed effects and interactions, starting with the significant effects of VOT duration and consonantal place (velar vs. non-velar). Whiskers represent the 95% confidence intervals (CIs), with smaller CIs indicating higher precision. OR values above 1.0 (to the right of the vertical gray line) indicate increased occurrence of ‘voiced’ responses. Values below 1.0 (to the left of the line) signal a decrease in ‘voiced’ responses. Values of 1.0 or approaching it (on or near the line) show that the rate of ‘voiced’ responses was unaffected. The further away an OR value is from 1.0, the stronger the causal relationship. As reflected in the figure, VOT duration had the strongest influence on participants’ judgments, with higher VOT values corresponding to a prominent decrease in the rate of ‘voiced’ response. In other words, tokens with little or no prevoicing were significantly more likely to be identified as ‘voiceless’. Furthermore, tokens with velars were more likely to be judged as ‘voiced’ than tokens with non-velars with the same VOT. The magnitude of the place effect was modest. The rest of the factors did not affect participants’ responses.

While the effects observed in Experiment 1 were in the expected direction, male and female voices may be expected to differ across multiple parameters that are potentially relevant for voicing judgments (e.g., spectral properties of the burst). Such differences may be more salient and may distract participants from focusing on VOT. This may have masked the true extent of the place of articulation effect and may also help explain the absence of significant effects of speaker sex and vocalic environment. To explore this possibility, a second identification experiment was conducted using resynthesized stimuli.

### 3.2. Experiment 2

In Experiment 2, participants listened to the original male voice and resynthesized female voice tokens. As in the first experiment, VOT duration was significant in the glmer model (β=−11.47;z=−13.89;p<0.001). Consonantal place (velar vs. non-velar) was also significant (β=0.36;z=2.77;p<0.01). Unlike Experiment 1, the effect of speaker sex was now marginally significant (β=−0.19;z=−1.67;p=0.095). All remaining fixed effects and interactions were not significant (all *p*s > 0.1). Full results of statistical modeling are provided in Table A3 in Appendix A.

**Figure 4:**
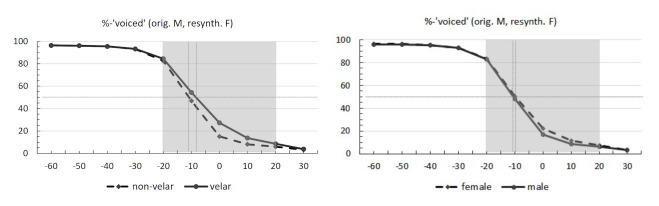
Identification curves for the original voice (male) and resynthesized voice (female) tokens identified as ‘voiced’ across 10 VOT levels. Left: non-velars (dashed line) vs. velars (solid line). Right: female voice (dashed line) vs. male voice (solid line). Thin dotted lines mark the 50% boundary. Statistical modeling was conducted on the area highlighted in grey.

Identification curves for the original and resynthesized voice tokens separated by consonantal place (velar vs. non-velar) and speaker sex (female vs. male) are provided in Figure 4. Classic S-shaped identification functions can be seen along the VOT continuum for all four categories. Tokens with less or no prevoicing consistently showed lower rates of ‘voiced’ responses. The 50% category boundary was at −11 ms for non-velars and −8 ms for velars. For both male and female voice tokens, the boundary was at around −10 ms. Full results for the mean rates of ‘voiced’ responses are given in Table A4 in Appendix A.

**Figure 5:**
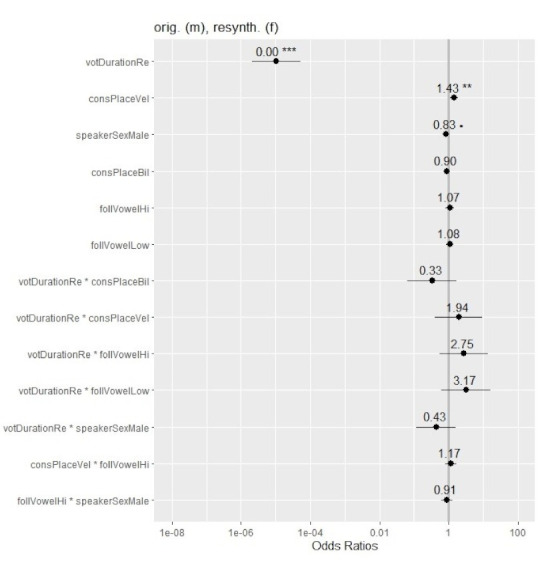
Odds ratios (ORs) of ‘voiced’ responses across the fixed effects and interactions in the glmer model for the original male and resynthesized female tokens in the −20 ms to +20 ms VOT range. Significance is shown with asterisks (‘***’: <0.001; ‘**’: <0.01; ‘.’: <0.1). Confidence intervals (CIs: 95%) are marked with whiskers.).

Figure 5 shows the odds ratios (ORs) for ‘voiced’ responses in Experiment 2. As can be seen in the figure, VOT duration had the strongest influence on participants’ identification of voicing in plosives, with the rate of ‘voiced’ responses decreasing as VOT increased. Place of articulation had a significant effect of lesser magnitude. Presence of a velar plosive was associated with higher odds of a ‘voiced’ response compared to tokens with bilabials and alveolars with the same VOT. These findings fully parallel the results for the effects of VOT duration and consonantal place in Experiment 1. Unlike the previous experiment, speaker sex showed a marginally significant effect of small magnitude, which was in the expected direction (i.e., more ‘voiceless’ responses for the male voice tokens in the −20 ms to +20 ms VOT range). Vowel height did not affect participants’ responses and none of the factors interacted. Implications of the current findings are discussed below.

## 4. Discussion

Overall, results from the two experiments indicated that the perceptual boundary for ‘voiced’ versus ‘voiceless’ categories is located within the negative VOT space in Russian, with values ranging between −12 ms to −8 ms. This is consistent with the previous findings for other prevoicing languages, such as Dutch and Spanish [5,33], and also matches the −16 ms VOT boundary that has been previously reported for Russian alveolars [35]. The small numeric difference between the current and previous findings for Russian is likely attributable to the differences in VOT continua, which is known to affect the location of the category boundary for voicing judgments [10].

The present study also observed an effect of consonantal place of articulation on participants’ responses. The effect was significant in both experiments. As stated in the Introduction, the AVC inhibits the production of voicing in the more posterior plosives [18]. Previous phonetic research has confirmed that the AVC plays a role in the Russian language, with initial velar plosives showing less prevoicing than non-velars [6,7]. The present perceptual findings reveal that the effect of place of articulation is not limited to production. As Russian speakers produce less prevoicing in velars, Russian listeners accept less closure voicing when classifying velars as ‘voiced’. This shows that the AVC can affect not only the initial production of VOT in plosives but also its subsequent perception. At the same time, the magnitude of the place effect is quite modest, which suggests that consonantal place of articulation plays only a limited role in perception.

Unlike the place of articulation, adjacent vowel height did not affect participants’ judgments in either experiment. In production, voicing duration is known to vary depending on vocalic context [23,24,26]. This has been attributed to coarticulation-related differences in the size of the oral cavity behind the oral constriction, which affects the extent to which air pressure can be lowered [23]; however, the vowel height effect has not been consistent in production. In some studies, vocal fold vibration is facilitated in high vowel contexts [23]. In other investigations, there are no differences across vowel heights [5] or the effect is in the opposite direction, with more prevoicing seen with non-high vowels but only for a subset of plosives and speakers [7]. This lack of consistency in production may in turn explain the absence of the effect in perception. In other words, Russian listeners do not have robust exposure to this type of asymmetry in their own speech and the speech of others, and they do not take the effect of vowel height into account when making voicing judgments.

For speaker sex, the effect was not significant in the first experiment when using original voice tokens that varied across multiple acoustic parameters. Differences between male versus female voice tokens became only marginally significant in the second experiment when acoustic variability was constrained. As noted earlier, production of voicing is thought to be easier for males due to a larger vocal tract [28], and several previous studies have observed an asymmetry in VOT duration or its frequency, with male voice tokens showing longer or more frequent closure voicing [5,6,25]; however, production findings for the role of speaker sex have been inconsistent. Some studies show more voicing for male speakers regardless of the phonetic context [6]. Other investigations report a significant effect for the high vowel context only [7] or even a reversed effect, with more closure voicing seen in female speech [29]. In the current study, listeners showed a possible tendency to perceive male voice tokens as voiceless more often than resynthesized female voice items with the same amount of VOT. However, the effect of speaker sex was not as consistent or prominent as the influence of place of articulation. As in the case of vowel height, this may be due to the lack of consistent exposure to a speaker sex asymmetry in production. The identification task paradigm may also not be sensitive enough to detect the effects of speaker sex or vowel height. Such influences may only exist at the earliest pre-lexical stages of processing, whereas identification responses are largely lexical and they even allow for post-lexical influences from contextual and background knowledge.

The current findings have implications for the role of phonetic influences in speech perception. A link between production and perception has long been advocated for in neurolinguistic and psycholinguistic literature, including the foundational aphasia studies [43] and several prominent theories of speech production and perception, such as the motor theory [44] and the direct-realist theory [45]. The strong version of the motor theory, for example, argues for both production and perception relying on the same phonetic information. In other words, knowing how a speech sound is produced and what kind of acoustic output is generated helps recognize the sound. This view is supported by the apparent activation of motor brain structures during not only articulation but also visual speech processing [46] and auditory perception [47,48]. A strong link between production and perception is also supported by the McGurk effect, which shows that listeners routinely integrate visual cues when identifying speech sounds [49]. It has also been demonstrated that silent articulation affects auditory processing [50] and that temporary disruption of the motor cortex can impair categorical perception [51]. At the same time, we know that individuals with permanent or temporary neurological or physiological impairments that affect speech production do not necessarily show impaired perception [52,53]. This includes previous experience with tracheostomy (intubation) for more than three months that leads to impaired production of vocal fold vibration but does not affect perception of voicing [54]. Thus, the relationship between production and perception cannot be causal [48,53,55]. This view is also shared by theoretical linguists who argue that phonological computation must disregard articulatory and acoustic detail (aka ‘substance free’ phonology) [56].

In the present study, listeners made voicing judgments for CV sequences with VOT in the ambiguous range. Since most of the syllables did not match existing Russian words, listeners could not simply rely on lexical knowledge, which is well known to affect perceptual responses [57]. They also did not have access to visual or somatosensory feedback. Listeners had to base their responses on acoustic differences in VOT alone or in combination with other cues to voicing that may be present in the token, such as f0 of the following vowel [58]. Perceptual responses showed effects of not only voicing duration but also consonantal place of articulation and, at a marginally significant level, speaker sex. This shows that certain VOT asymmetries exist in both production and perception; however, it cannot be inferred from the current results whether listeners accessed articulatory representations directly (as suggested by the motor theory) or whether they relied on perceptual representations that exist separately and contain fine acoustic detail (e.g., fully specified exemplars of voiced plosives that reflect production differences in voicing) [59,60]. The small magnitude of the place of articulation effect, marginal significance for speaker sex and the absence of differences across vowel heights further suggest that production and perception of voicing are not critically co-dependent, with knowledge of production asymmetries making only a limited contribution to the perceptual decision-making process for VOT. Phonemic identification tasks may also use a different set of mechanisms compared to non-laboratory comprehension [53], so this type of knowledge may only be called upon when other sources of information are limited or not available. Hence, the extent to which the AVC and related phonetic factors play a role in the perception of VOT in everyday communication remains to be determined. Future research will need to address this important question.

## 5. Conclusions

The present study examined the link between production and perception of the voicing contrast in a prevoicing language, focusing on the effects of consonantal place, adjacent vowel height, and speaker sex. Results of perceptual identification tasks revealed that place of articulation can affect not only the initial production of consonantal voicing but also its subsequent perception. The magnitude of the place effect was small, suggesting that it played only a secondary role in perception. Vowel height did not show a significant effect on perceptual judgments, in contrast to what has been reported in some production studies. Speaker sex showed marginal significance only, with the differences being in the expected direction. These findings are consistent with the theories that advocate for a limited link between production and perception.

## Data Availability

The original data are available upon request from the author.

## Abbreviations

The following abbreviations are used in this manuscript:

ANOVA	Analysis of variance
AVC	Aerodynamic voicing constraint
CV	Consonant–vowel sequence
OR	Odds ratio
VOT	Voice onset time

## Appendix A

**Table A1:** Generalized linear mixed model (Experiment 1, original voice tokens).

Fixed Effects	Estimate	Std. Error	z Value	Pr (>[z])
(Intercept)	−1.210611	0.118843	−10.187	<2 × 10${}^{-16}$ ***
votDurRe	−10.964394	0.813468	−13.479	<2 × 10${}^{-16}$ ***
consPlBil	−0.026452	0.113881	−0.232	0.81632
consPlVel	0.410131	0.128821	3.184	0.00145 **
follVlHi	−0.038413	0.162258	−0.237	0.81286
follVLow	0.029688	0.111306	0.267	0.78968
speakSexM	−0.149305	0.110787	−1.348	0.17776
votDurRe:consPlBil	−0.221077	0.831608	−0.266	0.79036
votDurRe:consPlVel	0.821977	0.800654	1.027	0.30459
votDurRe:follVHi	0.014414	0.822785	0.018	0.98602
votDurRe:follVLow	0.357623	0.802994	0.445	0.65606
votDurRe:speakSexM	−0.216758	0.665062	−0.326	0.74448
consPlVel:follVoHi	0.043204	0.199239	0.217	0.82833
follVHi:speakSexM	0.009037	0.190152	0.048	0.96209

Signif. codes: 0 ‘***’ 0.001 ‘**’ 0.01.

Formula: responseVoi votDurationRe * (consPlaceBil + consPlaceVel + follVowelHi + follVowelLow + speakerSexMale) + consPlaceVel * follVowelHi + speakerSexMale * follVowelHi + (1|itemName).

Control: glmerControl(optimizer = “bobyqa”, optCtrl = list(maxfun = 1 × 10${}^{5}$)).

Fit by maximum likelihood (Laplace Approximation) [glmerMod].

Family: binomial (logit).

**Table A2:** Mean rates of ‘voiced’ responses (%) across 10 VOT levels (Experiment 1, original voice tokens).

VOT	Non-Vel.	Velar	Non-Bilab.	Bilab.	Non-High	High	Non-Low	Low	Female	Male
−60	94.8	94.9	94.8	94.8	94.6	95.2	94.8	94.8	94.8	94.8
−50	94.1	94.4	94.5	93.7	94.3	94.0	94.0	94.7	94.3	94.1
−40	94.6	93.4	94.1	94.4	95.1	92.6	93.5	95.7	94.4	94.0
−30	91.6	92.1	91.9	91.4	91.4	92.4	91.3	92.7	91.8	91.7
−20	77.9	79.6	78.7	78.1	78.3	78.8	78.8	77.9	78.9	78.1
−10	44.0	51.9	48.5	42.9	48.1	43.8	46.5	46.9	47.4	45.9
0	13.2	25.4	18.9	13.9	16.8	18.2	16.5	18.8	19.5	15.0
10	6.7	12.2	9.5	6.6	8.3	9.0	8.9	7.8	9.7	7.3
20	6.0	7.3	6.8	5.7	6.8	5.8	6.2	7.0	6.4	6.5
30	3.8	4.4	4.2	3.7	3.8	4.6	4.2	3.7	3.9	4.1

**Table A3:** Generalized linear mixed model (Experiment 2, original and resynthesized voice tokens).

Fixed Effects	Estimate	Std. Error	z Value	Pr (>[z])
(Intercept)	−1.06217	0.12179	−8.722	<2 × 10${}^{-16}$ ***
votDurRe	−11.47183	0.82623	−13.885	<2 × 10${}^{-16}$ ***
consPlBil	−0.10882	0.11508	−0.946	0.34433
consPlVel	0.36090	0.13015	2.773	0.00556 **
follVHi	0.07211	0.16294	0.443	0.65810
follVLow	0.07651	0.11307	0.677	0.49859
speakSexM	−0.18795	0.11257	−1.67	0.09500.
votDurRe:consPlBil	−1.09387	0.84106	−1.301	0.19340
votDurRe:consPlVel	0.66496	0.80869	0.822	0.41092
votDurRe:follVHi	1.01264	0.83036	1.220	0.22265
votDurRe:follVLow	1.15350	0.82802	1.393	0.16360
votDurRe:speakSexM	−0.83819	0.67558	−1.241	0.21472
consPlVel:follVHi	0.15926	0.20108	0.792	0.42833
follVHi:speakSexM	−0.09564	0.19227	−0.497	0.61890

Signif. codes: 0 ‘***’ 0.001 ‘**’ 0.01.

Formula: responseVoi votDurationRe * (consPlaceBil + consPlaceVel + follVowelHi + follVowelLow + speakerSexMale) + consPlaceVel * follVowelHi + speakerSexMale * follVowelHi + (1 + votDurationRe|participantNum) + (1|itemName).

Control: glmerControl(optimizer = “bobyqa”, optCtrl = list(maxfun = 1 × 10${}^{5}$)).

Fit by maximum likelihood (Laplace Approximation) [’glmerMod’].

Family: binomial (logit).

**Table A4:** Mean rates of ‘voiced’ responses (%) across 10 VOT levels (Experiment 2, original and resynthesized voice tokens).

VOT	Non-Vel.	Velar	Non-Bilab.	Bilab.	Non-High	High	Non-Low	Low	Female	Male
−60	96.4	96.3	96.3	96.4	96.9	95.2	96.1	96.9	96.6	96.1
−50	96.1	96.1	96.3	95.7	95.9	96.3	95.8	96.6	96.2	95.9
−40	95.4	95.1	95.4	95.1	95.1	95.9	94.9	96.1	95.3	95.4
−30	93.2	93.2	93.6	92.4	93.3	92.9	92.8	94.0	93.2	93.2
−20	82.5	84.4	83.3	82.8	83.2	83.1	83.5	82.4	83.3	83.0
−10	46.9	54.3	50.9	46.3	49.8	48.6	50.1	48.0	50.6	48.2
0	15.2	27.3	21.2	15.3	19.3	19.1	18.4	21.0	21.9	16.7
10	8.2	13.7	11.1	7.9	9.4	11.2	10.2	9.6	11.6	8.4
20	5.7	8.6	7.7	4.6	6.4	7.0	6.2	7.4	7.3	6.0
30	3.2	3.8	3.6	3.2	3.7	3.0	3.4	3.7	3.4	3.6
